# Cedratvirus, a Double-Cork Structured Giant Virus, is a Distant Relative of Pithoviruses

**DOI:** 10.3390/v8110300

**Published:** 2016-11-03

**Authors:** Julien Andreani, Sarah Aherfi, Jacques Yaacoub Bou Khalil, Fabrizio Di Pinto, Idir Bitam, Didier Raoult, Philippe Colson, Bernard La Scola

**Affiliations:** 1 Unité de Recherche sur les Maladies Infectieuses et Tropicales Emergentes (URMITE), UM63 CNRS 7278 IRD 198 INSERM U1095, Facultés de Médecine et de Pharmacie, 13385 cedex 05 Marseille, France; miaguiabidou@gmail.com (J.A.); aherfi.s@gmail.com (S.A.); boukhaliljacques@gmail.com (J.Y.B.K.); didier.raoult@gmail.com (D.R.); philippe.colson@univ-amu.fr (P.C.); 2 Institut Hospitalo-Universitaire (IHU) Méditerranée Infection, Pôle des Maladies Infectieuses et Tropicales Clinique et Biologique, Fédération de Bactériologie-Hygiène-Virologie, Centre Hospitalo-Universitaire Timone, Assistance Publique—Hôpitaux de Marseille,13005 Marseille, France; tem.timone@gmail.com; 3 Laboratoire Biodiversité et Environnement: Interactions Génomes, Faculté des Sciences Biologiques Université des Sciences et de la Technologie Houari Boumediene, BP 32 EL ALIA 16111 Bab Ezzouar Alger, Algeria; idirbitam@gmail.com

**Keywords:** giant viruses, Cedratvirus, *Pithovirus*, viral family, double-cork, co-culture, *Acanthamoeba*

## Abstract

Most viruses are known for the ability to cause symptomatic diseases in humans and other animals. The discovery of *Acanthamoeba polyphaga mimivirus* and other giant amoebal viruses revealed a considerable and previously unknown area of uncharacterized viral particles. Giant viruses have been isolated from various environmental samples collected from very distant geographic places, revealing a ubiquitous distribution. Their morphological and genomic features are fundamental elements for classifying them. Herein, we report the isolation and draft genome of Cedratvirus, a new amoebal giant virus isolated in *Acanthamoeba castellanii*, from an Algerian environmental sample. The viral particles are ovoid-shaped, resembling *Pithovirus sibericum*, but differing notably in the presence of two corks at each extremity of the virion. The draft genome of Cedratvirus—589,068 base pairs in length—is a close relative of the two previously described pithoviruses, sharing 104 and 113 genes with *P. sibericum* and *Pithovirus massiliensis* genomes, respectively. Interestingly, analysis of these viruses’ core genome reveals that only 21% of Cedratvirus genes are involved in best reciprocal hits with the two pithoviruses. Phylogeny reconstructions and comparative genomics indicate that Cedratvirus is most closely related to pithoviruses, and questions their membership in an enlarged putative *Pithoviridae* family.

## 1. Introduction

For 13 years, high-throughput culture isolation strategies and genomic analyses have led to the discovery of a considerable diversity of amoeba-infecting giant viruses. These discoveries have revolutionized the definition of viruses, and have raised questions on the role of eukaryotic viruses in the evolutionary history of living organisms [1]. The description of the giant viruses with icosahedral capsids, including *Acanthamoeba polyphaga mimivirus*, the pioneer isolate [2], then of marseilleviruses [3] and faustoviruses [4], was followed by the description of viruses with atypical virion morphologies and ultrastructures, including the pandoraviruses [5,6] *Pithovirus sibericum* [7] and *Mollivirus sibericum* [8]. These two latter viruses were isolated from 30,000-year-old permafrost, demonstrating a conserved infectious effect for amoebas [7,8]. The recent isolation of another virus, *Pithovirus massiliensis*, from sewage, offered the opportunity to analyze the evolution between the modern and fossil genomes [9]. Surprisingly, the estimated mutation rate was found to be lower than RNA viruses and some DNA viruses, and these genomes exhibited stability comparable to that of prokaryotic genomes. Morphologically, previously described pithoviruses have a single cork at the apex region, suggesting a polarity essential for the infection of amoebas [7]. We describe herein a new giant virus isolate, morphologically and genetically close to pithoviruses, which was named Cedratvirus because of its lemon-shape (*Citrus medica*). Cedratvirus has an ovoid virion and a cork at each apex. Genomic analyses revealed that Cedratvirus is most closely related to pithoviruses and may expand the putative *Pithoviridae* family, but displays new features, such as a different set of genes involved in the biosynthesis of sugars and amino-acids, and a distant relationship with the two previously described pithoviruses.

## 2. Materials and Methods

### 2.1. Culture and Isolation Procedures

Twenty-four samples were collected in October 2015 from Constantine, Annaba, Taref, El-Kala and Oran, in Algeria. We performed a classic co-culture method associated with presumptive identification by flow cytometry [9,10]. *Acanthamoeba castellanii* strain Neff was used as cell support. The amoebas were harvested after 48 h in culture in Peptone Yeast Extract Glucose medium (PYG, home made) when they reached a concentration of 5.10^5^ amoebas/mL. Cells were rinsed twice in Page’s Amoeba Saline (PAS, home made) and pelleted at 700× *g* for 10 min. Afterwards, the amoebas were re-suspended in the starvation medium [10] at a concentration of 5.10^5^ amoebas/mL. Antibiotic and antifungal mixture with vancomycin (10 μg/mL), ciprofloxacin (20 μg/mL), imipenem (10 μg/mL) and voriconazole (20 μg/mL) were added to the suspension in order to decrease or eliminate bacterial or fungal contaminations. Cell suspension was then distributed in a 48-well plate at the level of 250 μL per well, the samples were vortexed and 50 μL were added to each well. The rest of the wells served as negative control by adding 50 μL of PAS. The plate was incubated at 30°C for four days. The cytopathic effect was monitored under optical inverted microscope. This co-culture was repeated twice in the same order. This technique is well detailed and discussed in the work of Bou Khalil et al. [11].

### 2.2. End-Point Dilution and Centrifugation Methods

In order to obtain a pure viral population, viral supernatant was centrifuged at 4000× *g* for 15 min. The pellet was re-suspended with 1 mL of PAS three times. Finally, fresh *A. castellanii* cells were inoculated with the re-suspended pellet.

End-point dilution was performed in order to clone the virus before its production, and to separate viral sub-populations. For that, we successively inoculated diluted viral supernatant at a dilution factor of 10 on *A. castellanii*. End point dilution was assessed for five days, and the lysis was controlled by electron microscopy and flow cytometry.

### 2.3. Cytometry Applications

After lysis detection, we managed to identify the lytic agent by applying the protocol described by Bou Khalil et al. [12] using SYBR Green I nucleic acid gel stain (Molecular Probes, Life Technologies, USA) to process 10 μL of the culture supernatant through flow cytometry. This technique enabled us to differentiate putative amoebal lytic agents based on previous gating of known giant viruses already characterized by flow cytometry. Once detected, and in the case of viral mixture, the end point dilution and centrifugation methods failed to separate and purify each viral population. Therefore, we applied a new sorting technique adapted in our lab and under review to purify Cedratvirus by flow cytometry using a BD FACS JAZZ^®^ sorter (BD biosciences, Rungis, France) [13]. Purity control was assessed using electron microscopy.

With respect to the production and the purification, briefly, 15 infected flasks of 150 cm^2^ (Corning^®^, NY, USA) were pelleted using the Beckman coulter^®^ centrifuge Avanti® J-26 XP (Beckman, France) at 14,000× *g* for 30 min [9]. A 25% sucrose gradient was used for the final purification step. After finalizing the production, we proceeded to genome sequencing.

### 2.4. Electron Microscopy and Infectious Cycle Description

Negative staining was performed on the fixed supernatant from co-culture. We deposited 5 μL onto the glow-discharged grid for 20 min at room temperature. The dried grid was contrasted with a small drop of 1% ammonium molybdate for 10 seconds and the grid was then observed on a Tecnai G20 (FEI, Germany).

For the infectious cycle description, a pure viral suspension was used to infect four flasks of 30 mL, each containing amoeba suspension of 5.10^5^ amoebas/mL, at a multiplicity of infection (MOI) of 10. After 30 min of post infection incubation, the amoeba monolayer was washed three times with PAS buffer to eliminate non-internalized viruses. This time point was designated as H0. A total of 10 mL of the infected cultures were distributed into new culture flasks incubated at 30 °C. A culture flask containing a non-infected flask of amoeba was used as the negative control. At 0, 2, 4, 6, 8, 10, 12, 16, 20, 24, and 28 h post-infection (hpi), each culture flask corresponding to a specific time point was centrifuged at 720× *g* for 10 min, and the pellets were fixed for the transmission electron microscopy procedures. With regards to these, *A. castellanii*-infected cells were recovered and pelleted for 10 min at 5000× *g*. The pellet was re-suspended in 1 mL of phosphate-buffered saline (PBS) with 2% glutaraldehyde–0.1 M cacodylate and incubated for at least 1 h at 4 °C. Each pellet was then washed three times with 0.1 M cacodylate–saccharose and resuspended in the same buffer. After re-pelleting, each sample was then embedded in Epon resin by using the following standard method: 1 h of fixation in 1% osmium tetroxide, two washes in distilled water, dehydration in increasing successive ethanol concentrations (30%, 50%, 70%, 96%, and 100% ethanol), and embedding in Epon-812. Ultrathin (70 nm) sections were post-stained with 5% uranyl acetate and lead citrate [14]. Electron micrographs were obtained on a Tecnai G20 F20 TEM (FEI, Germany) operated at 200 keV. ImageJ (https://imagej.nih.gov/ij/) software was used to determine particle size at the different time points of the cycle.

### 2.5. Genome Sequencing

The genomic DNA of Cedratvirus was sequenced using the MiSeq Technology (Illumina Inc., San Diego, CA, USA) with two methods paired-end with the Nextera XT DNA sample prep kit (Illumina), and mate pair with the Nextera Mate Pair sample prep kit (Illumina). The extracted genomic DNA was quantified by the Qbit DNA HS Assay kit (Life technologies, Carlsbad, CA, USA) at 513.3 ng/μL and was barcoded in order to be pooled along other projects.

To prepare the paired-end library, dilution was performed to obtain an input of 1 ng of genomic DNA. The “tagmentation” step fragmented and tagged the DNA, and then limited-cycle polymerase chain reaction (PCR) amplification (12 cycles) completed the tag adapters and introduced dual-index barcodes. The library profile was validated on an Agilent 2100 Bioanalyzer (Agilent Technologies Inc., Santa Clara, CA, USA) with a DNA High sensitivity LabChip, and the fragment size was estimated to be approximately 0.5 kb. After purification on AMPure XP beads (Beckman Coulter Inc., Fullerton, CA, USA), the libraries were normalized on specific beads according to the Nextera XT protocol (Illumina). Normalized libraries were pooled for sequencing on the MiSeq. Automated cluster generation and paired-end sequencing with dual index reads were performed in a 2 × 250-bp run. Total information of 9.0 Gb was obtained from a 1019 k/mm^2^ cluster density, with cluster passing quality control filters of 90.2% (17,374,744 passed filtered clusters). Within this run, the index representation for Cedratvirus was determined to be 3.85%. The 669,188 paired-end reads were trimmed and filtered according to the read qualities.

The mate pair library was prepared with 1.5 μg of genomic DNA using the Nextera mate pair Illumina guide. The genomic DNA sample was simultaneously fragmented and tagged with a mate pair junction adapter. The profile of the fragmentation was validated on an Agilent 2100 Bioanalyzer (Agilent Technologies Inc., Santa Clara, CA, USA) with a DNA 7500 LabChip. The optimal size of obtained fragments was 6.13 kb. No size selection was performed and 631.9 ng of tagmented fragments were circularized. The circularized DNA was mechanically sheared to generate small fragments with an optimal size of 970 bp on the Covaris S2 device in T6 tubes (Covaris, Woburn, MA, USA). The library profile was visualized on a High Sensitivity Bioanalyzer LabChip (Agilent Technologies Inc., Santa Clara, CA, USA) and the final concentration library was measured at 34.04 nmol/L. The library was pooled with 11 other projects normalized at 2 nM; this pool was denatured and diluted at 15 pM before sequencing in a 2 × 250-bp run. The total information of 7.9 Gb was obtained from a 863 K/mm^2^ cluster density, with cluster passing quality control filters of 94.0% (15,627,076 passed filter clusters).

### 2.6. Genome Assembly

Mate pair and paired-end reads were trimmed using CLC Genomics Workbench v7.5 (http://www.clcbio.com/blog/clc-genomics-workbench-7-5/). De novo assembly of 1,519,052 reads was done using 64 word size and 50 bubble size parameters. We obtained one contig of 423,175 base pairs (bp) and a second contig of 163,805 bp. To fill gaps, specific PCR primers were designed by using Primer-BLAST [15]. We tried the assembly with a different k-mer parameter, more adapted to the size of our reads. Successively, we used 80 and 92 k-mer sizes with the Abyss program [16] and made a scaffolding using SSPACE software [17] on each result. With these two assembly methods and Sanger sequencing products we obtained a single scaffold.

### 2.7. Study of the Genome Organization and Genome Annotation

Repeats in the genome were investigated by complementarities from EMBOSS software (http://emboss.bioinformatics.nl/cgi-bin/emboss/palindrome) with 200 nucleotides for maximum length of palindromic sequences and by CRISPRFinder [18] with standard parameters. Open reading frames were predicted by GeneMarkS [19] and the genome was deposed under BioProject No. PREJ15450 in the EMBL-EBI database. The tRNA genes were searched for by using tRNAscan-SE and ARAGORN softwares [20,21]. Predicted proteins of less than 50 amino acids in length were discarded. The remaining proteins ranging from 50 to 99 amino acids in length were discarded if they did not have a hit in the BLASTp search in the NCBI GenBank non-redundant protein sequence database. Predicted proteins longer than 99 amino acids were kept for the analysis. A BLASTp search against the NCBI non-redundant protein sequence database and against predicted proteins from *P. massiliensis* was performed. Homology was considered significant if the e-value was lower than 1 × 10^−3^. A BLASTp search was also computed with the same parameters against the clusters of orthologous groups of proteins of the nucleocytoplasmic large DNA virus (NCVOGs) [22]. A cladistic representation based on the presence/absence of each NCVOG was performed by MultiExperiment Viewer (MeV) [23]. Conserved domains and putative functions of viral proteins were predicted by comparative genomics through a BLASTp search against the NCBI non-redundant protein sequence database and by using InterPro v58.0 (https://www.ebi.ac.uk/interpro/); results merged with specific Delta-BLAST results [24]. We explored bona fide orthologous genes by using Proteinortho v4 [25] with 50% coverage and 30% amino acid identity and an e-value of 1 × 10^−2^ as significance thresholds. Paralogous genes were predicted by the BLASTClust program [26] with 70% coverage and 30% amino acid identity as thresholds.

### 2.8. Phylogenetic Analyses

The MUSCLE program [27] was used to align amino acid sequences. The FastTree program [28] was computed with standard parameters (using the Jones-Taylor-Thornton (JTT) model for amino acid substitution) by using the maximum likelihood method with 1000 bootstrap replicates. Then, phylogenetic trees were visualized by using iTOL v3 online [29].

## 3. Results

### 3.1. Isolation of Cedratvirus

Lysis was monitored using light microscopy and cytopathic effect was observed in four wells. The wells that presented lysis were controlled by sampling 40 μL from the supernatant on agar plates in order to exclude bacterial contaminations. In the end, no bacterial growth was observed after five days. Flow cytometry presumptive identification was done on the lysis wells supernatant. We followed our protocol based on the gating strategy [12] and we detected the presence of a mix of a mimivirus and new unidentified particles population in one sample. The negative staining performed on this sample culture supernatant revealed the presence of new doubled cork shaped particles corresponding to the newly named Cedratvirus. After the failure of both centrifugation and end-point dilution methods, and in order to obtain a pure viral population containing only Cedratvirus, we applied a new sorting method adapted in our lab and under revision to sort the pure population of Cedratvirus. The pure sorted fraction was re-cultured for production and sequencing.

### 3.2. Ultrastructure and Replicative Cycle of Cedratvirus

**Figure 1:**
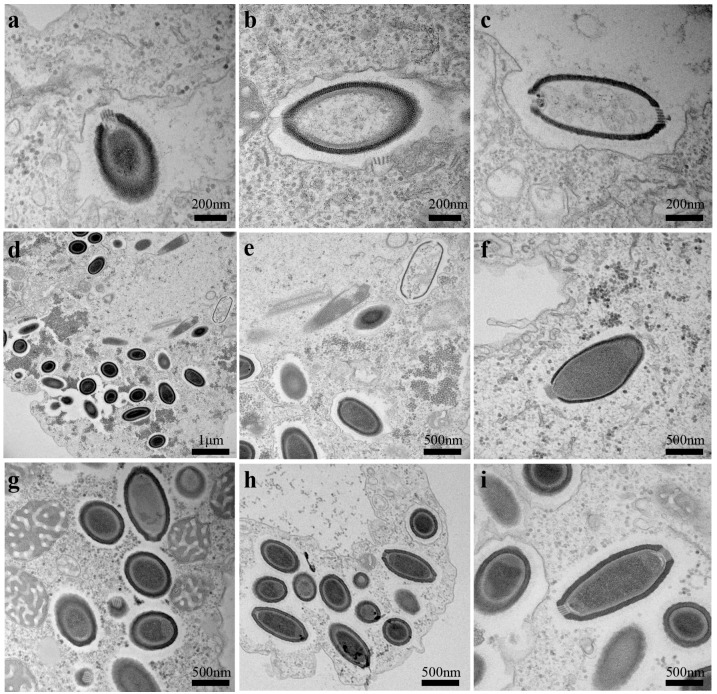
Ultrathin electron microscopy imaging of the Cedratvirus replication cycle in *Acanthamoeba castellanii*. (**a**) Neo-phagocystis particle in vacuole; (**b**) Channel formation in first step of infection; (**c**) Empty particle 2 h post-infection (hpi) into a vacuole; (**d**–**h**) Various stages of concomitant Cedratvirus between 6 and 8 hpi in the cytoplasm of the amoeba; (**i**) Typical mature particle with the two corks.

The Cedratvirus virions have a length ranging from 1 μm to 1.2 μm, and a maximal diameter of 0.5 μm. The replicative cycle starts with the internalization of the viral particle by phagocytosis. The monolayer surrounding the virion displays two variable levels of thickness at different infection time points. In the early period of infection (Figure 1a–c), the layer measures only 40 ± 5 nm (*n* = 10), then reaches 55 ± 5 nm (*n* = 12) (Figure 1d–i) at the stage of mature virions. This variation may be a result of the phagosomal process of the virions in phagosomes post-internalization, and their progressive degradation in the vacuoles after the injection of the genomic DNA into the amoebal cytoplasm. Then, we also observed the same channel for the DNA as previously described for *P. sibericum* (Figure 1a,b). Empty particles could be seen after the DNA injection and we were able to detect particles with two-sided corks, but only one cork responsible for the DNA delivery (Figure 1c). The virus has an infectious cycle typical for giant viruses regarding the eclipse phase, with no viral particles detected at 4 hpi and the presence of a virus factory. Six to eight hpi, mature virions are well-detected inside amoebas, with replication still on-going for some others (Figure 1d,e). Partial burst cell started at 10 hpi and complete amoebal lysis was observed after 24 hpi, with a MOI of 10:1 viruses per amoeba. Hence, the major differences with previously described pithoviruses are the presence of two corks in virions (Figure 1h,i) and a smaller grid structure for both corks, compared with the one observed in *P. sibericum*, as revealed by observation of transversal electron microscopy sections.

### 3.3. Genome Analysis

The Cedratvirus A11 genome is a double-stranded circular DNA molecule of 589,068 bp in length with a GC% of 42.6%. At first sight (Table 1), the genome appears smaller, by approximately 21 and 97 kilobase pairs (kbp) compared with *P. sibericum* and *P. massiliensis*, respectively. However, uneven read distribution and abnormally large insert as described for *P. sibericum* linear genome version were not observed for the Cedratvirus genome. In addition, no large palindromic repeats were identified by EMBOSS Palindrome. Nevertheless, the 20 gaps of the Cedratvirus draft genome were flanked by similar short sequences. The CRISPRFinder program detected 27 areas of repeats in the genome.

The Cedratvirus genome was predicted to encode 574 proteins. This represents, when combined with a shorter genome size, a greater coding capacity than for *P. sibericum* and *P. massiliensis*, with 107 and 54 more genes, respectively (Table 1). No tRNA was detected with the two methods applied. A total of 397 Cedratvirus genes (69.2%) had at least one homolog in the NCBI Genbank database, whereas 30.8% (177 proteins) had no significant hit, thus being classified as encoded by ORFan genes with an average length of 556 ± 247 nucleotides. The 397 proteins with known homologs showed the greatest homologies with proteins from 258 viruses, 108 eukaryotes and only 31 prokaryotes. As much as 84.1% of the best viral hits were with pithoviruses, with 107 proteins (40% of the best viral hits) and 116 proteins (43%) having best matches with *P. sibericum* and *P. massiliensis*, respectively. A total of 44 proteins had their best homolog in the genomes of other giant viruses, in particular, 10 with marseilleviruses. A total of 62% of the 108 best eukaryotic hits were from *Micromonas pusilla* strain CCMP1545 (40 proteins, or 37%, of the eukaryotic hits), *A. castellanii* str. Neff (13 proteins, or 12%) and *Ectocarpus siliculosus* (14 proteins, or 13%). Even if it is not rare to find a large proportion of protist-related genes in giant viruses, the numbers found for the two green algae were increased here by a high number of paralogs as confirmed by BLASTClust detection (Table S1, line 1), representing a large family of genes with hypothetical functions and ankyrin repeats. In contrast, for *A. castellanii*, the high number of best matches is probably linked with host specificity and probably results from horizontal gene transfers, and these proteins could be beneficial for the virus during infection. Only 31 proteins of Cedratvirus had a prokaryotic homolog as a best hit, including seven from *Bacillus* spp. and two from *Paenibacillus* spp. In addition, only 242 of the 574 Cedratvirus proteins had a predicted function according to the comparative genomic analyses.

Various genes from our annotated gene set seem to be related to new, unclear metabolic processes, for example to rhamnose synthesis. However, no complete pathway was identified. In addition, many Cedratvirus gene functions are new among giant viruses, especially among pithoviruses. For instance, aromatic amino acid synthesis genes were found, including an endoribonuclease L-PSP/chorismate mutase-like encoding gene (locus tag BQ3484_354) without a homolog in other DNA viruses, while a D-3-phosphoglycerate dehydrogenase, type 2 gene (BQ3484_10) had distant homologs in some Chlorella viruses (a D lactate deshydrogenase). In addition, many glycosyltransferases, acylCoA-transferases, and transferases were present in pithoviruses, but were more abundant in Cedratvirus. Duplicate genes are present for RNA polymerase II subunit 1, as in *P. sibericum* and *P. massiliensis*, while Cedratvirus also has two copies of genes annotated as ribonuclease III (BQ3484_182 and BQ3484_555). Cedratvirus has one gene that has a ribonuclease H (BQ3484_454) as a distant homolog.

### 3.4. Cedratvirus, Pithovirus sibericum and Pithovirus massiliensis: Three Members of a Putative Extended Family

**Figure 2:**
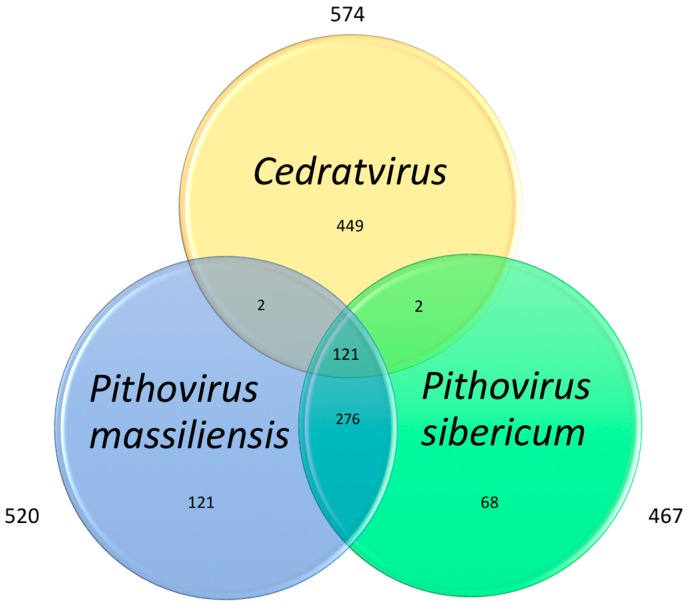
Co-orthologous genes representing the current putative *Pithoviridae* family.

Analysis of the best reciprocal hits (Figure 2) for the gene repertoires of Cedratvirus and the two previously described pithoviruses revealed that Cedratvirus shares about 21% of its gene content with these two pithoviruses, representing 121 core genes. A total of 52.9% of this core genome consists of ankyrin repeat-containing proteins (eight sequences) and hypothetical proteins (56 sequences). We also noted the presence in the core genome of essential genes involved in the replication (i.e., DNA polymerase, DNA-dependent RNA polymerase subunits, helicases) and of genes associated with DNA reparation (i.e., DNA repair exonuclease, alkylated DNA repair protein).

**Figure 3:**
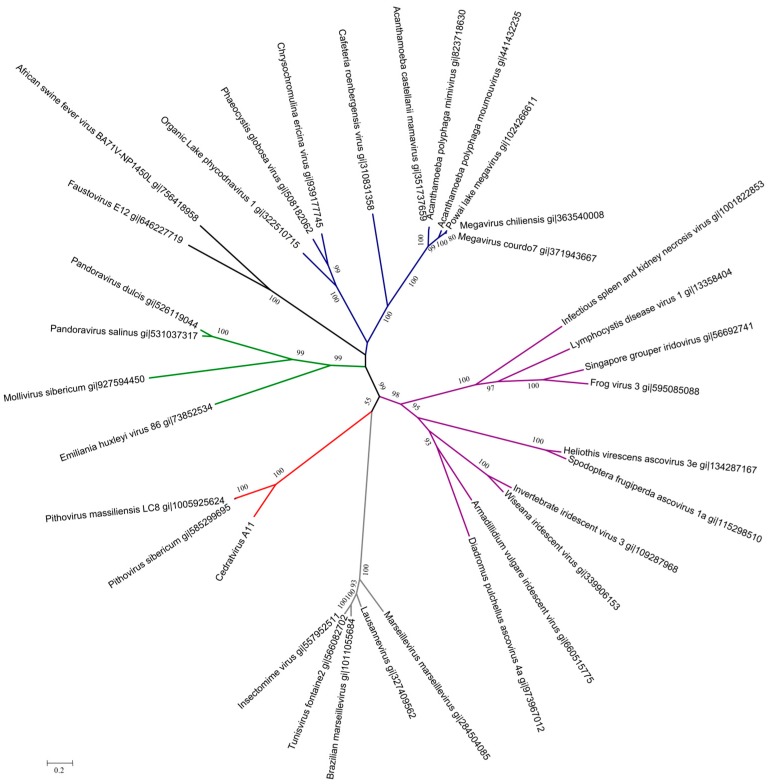
Unrooted tree based on DNA-dependent RNA polymerase II subunit 1 amino acid sequences; bootstrap values inferior to 0.5 (50%) were collapsed, branch length was applied; colors indicate family or viral groups: blue was used for *Mimiviridae* and an extended group including *Phaeocystis globosa* virus; purple for *Ascoviridae*-*Iridoviridae*; grey for *Marseilleviridae*; red for Cedratvirus and pithoviruses; green for *Phycodnaviridae*, pandoraviruses, and *Mollivirus sibericum* and black color for African swine fever virus and Faustovirus.

Phylogenetic reconstructions based on four core genes, namely, the DNA polymerase B family, the DNA-dependent RNA polymerase II subunits 1 and 2, and the VV-A18 helicase, revealed that Cedratvirus is a close relative of the other pithoviruses, which raises questions about its classification as a new member of a putative *Pithoviridae* family (Figure 3 and Figures S1–S3). Congruently, the cladistic representation based on the presence/absence of NCVOGs in the gene contents showed the relationship between Cedratvirus and previously described pithoviruses (Figure S4).

Regarding the NCVOG core genes, we found some conserved genes present also in some other nucleocytoplasmic large DNA viruses i.e D5 helicase-primase (NCVOG0023), topoisomerase II (NCVOG0037), mRNA capping enzyme (NCVOG1117), ribonucleotide reductase (NCVOG1353 and NCVOG0276) and a divergent major capsid protein (NCVOG0022). However, some genes could not be found such as the A32-like packaging ATPase (NCVOG0249), which was also absent in pithoviruses. Finally, Cedratvirus will bring in new categories in the NCVOG gene set.

## 4. Discussion

Cedratvirus is a new, extraordinary giant virus. Its remarkable features include its morphological properties, the size of its virions, the two cork virions compared to one cork in previously described pithoviruses, and also its gene content with a high proportion of ORFans (about one third). It also has greater coding density and GC% compared to these pithoviruses, and a large set of 449 new genes, not detected in *P. sibericum* and in *P. massiliensis*, even when we used a low value for significance threshold. Moreover, 58% of the gene content of Cedratvirus consists of hypothetical proteins, representing unknown functions. In our current understanding, Cedratvirus is the first virus with two apertures in its virions. We are not yet able to determine the exact capacities or benefits this double-cork might offer during replication. Regarding data from phylogenomic analyses, Cedratvirus has a genome similar in size to the genomes of the two pithoviruses, but the gene contents are clearly different in many instances. Moreover, the genome organization of Cedratvirus does not include the same large palindromic repeats in the intergenic regions as observed in *P. sibericum*. Phylogeny reconstructions showed that Cedratvirus is most closely related to pithoviruses, albeit distantly, but this relies on a very limited set of highly conserved genes shared by Cedratvirus, pithoviruses and some other giant viruses.

In a recent review [11] we discussed the characteristics of concomitant worldwide isolates of different giant viruses in the same sample. Here, the end-point dilution and centrifugation methods failed to isolate a pure strain of Cedratvirus. We observed again a fitness propagation in favor of the mimivirus replication. Therefore, the FACS sorter is more sensitive for detection and more efficient in the purification and cloning process, at least in the case of a viral mixture with different fitness where it is important to sort and isolate the virus of slower replication.

Fisher recently proposed to classify giant viruses using two other genes that encode a MutS protein and an asparagine synthetase [30], but neither Cedratvirus nor pithoviruses possess these genes. When considering members of the four distinct lineages described in *Marseilleviridae*, the viral family was found to be the closest to pithoviruses [31,32]. Some degrees of genomic conservation, and highly similar GC%, are noted between the *Marseilleviridae* members. In contrast, Cedratvirus and pithoviruses appear to be more genetically divergent. Thus, differences in their gene content is comparable to those described between *Phaeocystis globosa* virus PgV-16T [33], *Megavirus chiliensis*, and *Cafeteria roenbergensis* virus (CroV) (PgV-16T and CroV viruses are currently considered as composing an extended family of the *Mimiviridae*). Indeed, about one-third of the genes of PgV-16T were not found in *M. chiliensis* and CroV. Therefore, in accordance with the description for mimiCOGs by Yutin et al. [34], there is a similar level of divergence between our results and what has been observed in the *Mimiviridae* family between giant amoeba mimiviruses and distantly related viruses infecting marine eukaryotic hosts. Furthermore, the presence/absence patterns of genes conserved among giant viruses also indicate that Cedratvirus is most closely related to pithoviruses.

Taken together, the culture, genomic and morphological data suggest that Cedratvirus is most closely related to pithoviruses. However, Cedratvirus is a new TRUC (Things Resisting Uncompleted Classification). The strength of genetic links with these viruses and a putative *Pithoviridae* family needs to be clarified in further studies, possibly with new additional closely-related members. Nevertheless, the characterization of Cedratvirus broadens the knowledge of the diversity of giant amoeba viruses. It indicates that an improved set of criteria is needed for a clear definition of genera and families in giant viruses. The characterization of other close virus relatives of Cedratvirus, *P. sibericum* and *P. massiliensis* will allow a better understanding of the relationship between these viruses among other giant viruses.

## Figures and Tables

**Table 1:** Overview of Cedratvirus and *Pithovirus* properties.

	Cedratvirus A11	*Pithovirus sibericum*	*Pithovirus massilliensis*
Genome size (bp)	589,068	610,033	686,015
GC %	42.6	35.8	35.4
Genes	574	467	520
tRNA	0	0	0
Coding capacity (%)	78.5	69.0	64.0
Global morphology	Ovoid particle with double corks	Ovoid particle with a single cork	Ovoid particle with a single cork

## Supplementary Materials

The following are available online at www.mdpi.com/1999-4915/8/11/300/s1, Table S1: Top 12 most represented groups of paralogous genes as determined by the BLASTClust program, Figure S1: Unrooted tree based on family B DNA polymerase amino acid sequences, Figure S2: Unrooted tree based on DNA-dependent RNA polymerase II subunit 2 amino acid sequences, Figure S3: Unrooted tree based on VV-A18 helicase amino-acid sequences, Figure S4: Cladogram based on NCVOGs.
