# Supplementary material for: Cedratvirus, a Double-Cork Structured Giant Virus, is a Distant Relative of Pithoviruses
Source: Viruses. 2016 Nov 3;8(11):300. doi: 10.3390/v8110300 (PMC5127014; doi:10.3390/v8110300)
Supplement: Supplementary file 1 [file viruses-08-00300-s001.docx]

Supplementary Materials: Cedratvirus,
a Double-Cork Structured Giant Virus,
is a Distant Relative of Pithoviruses

Julien Andreani, Sarah Aherfi, Jacques Yaacoub Bou Khalil, Fabrizio Di Pinto, Idir Bitam, Didier Raoult, Philippe Colson and Bernard La Scola

**Table S1.** Top 12 most represented groups of paralogous genes as determined by the BLASTClust program (https://toolkit.tuebingen.mpg.de/blastclust).

| **Protein Families** | **Numbers of Genes** |
| --- | --- |
| *Ankyrin repeat and pseudo-ankyrin repeat-containing proteins* | 51 |
| *Hypothetical proteins* | 16 |
| *F-box containing proteins* | 14 |
| *Cyclin dependent kinases* | 10 |
| *Collagen triple helix repeat-containing proteins/BclB proteins* | 9 |
| *Hypothetical proteins* | 9 |
| *F-Box containing protein* | 7 |
| *Hypothetical proteins/ankyrin repeat-containing proteins* | 5 |
| *Acyl-CoA N-acyltransferases* | 5 |
| *Protein kinases* | 5 |
| *Hypothetical proteins* | 4 |
| *Ankyrin repeat containing proteins* | 4 |


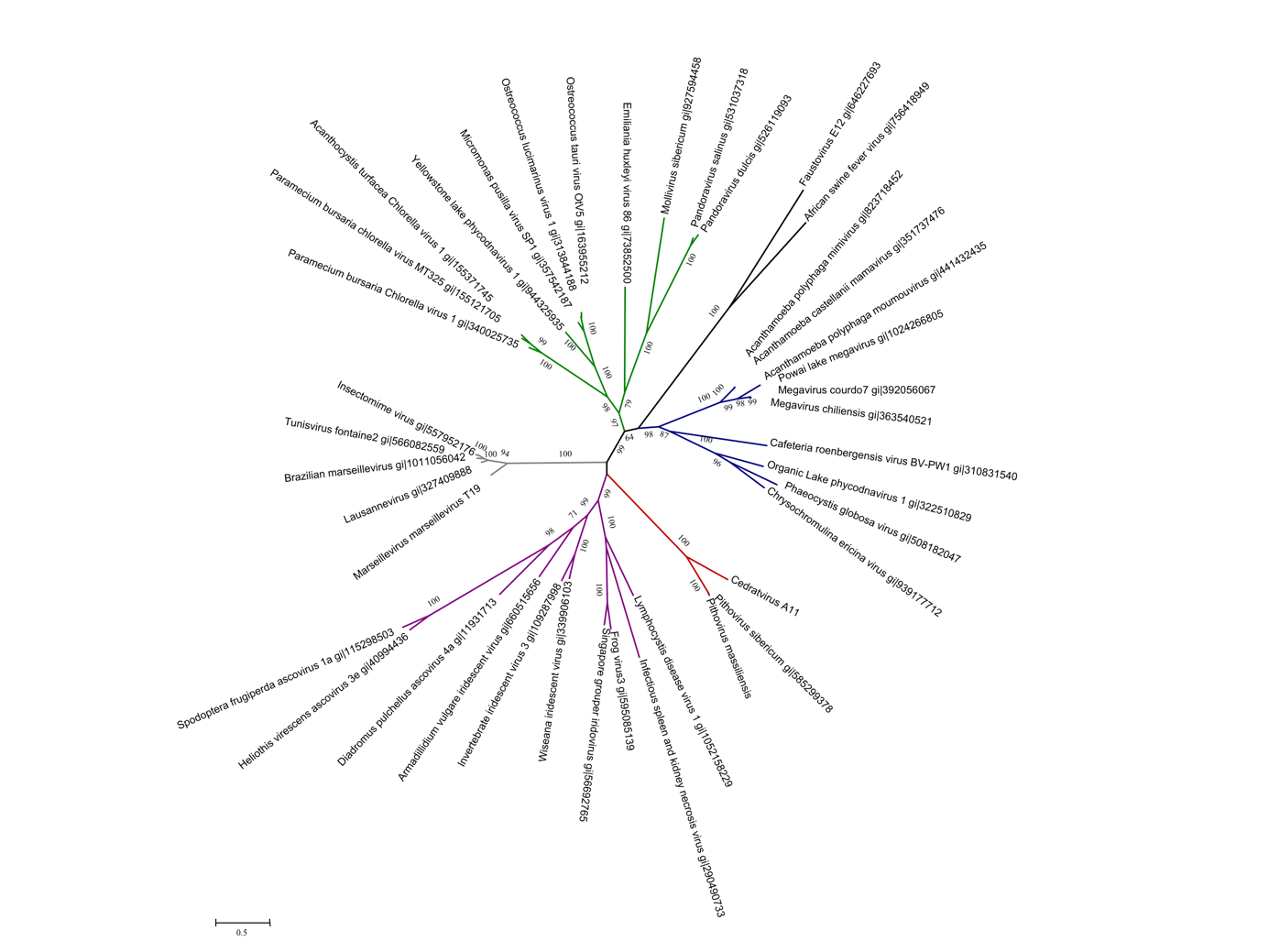


**Figure S1.** Unrooted tree based on DNA polymerase B family amino acid sequences; bootstrap values inferior to 0.5 (50%) were collapsed, branch length was applied; colours indicate family or viral groups: blue was used for *Mimiviridae* and an extended group including *Phaeocystis globosa virus*; purple for *Ascoviridae-Iridoviridae*; grey for *Marseilleviridae*; red for Cedratvirus and pithoviruses; green for *Phycodnaviridae*, pandoraviruses, and *Mollivirus sibericum* and black color for African swine fever virus and Faustovirus.


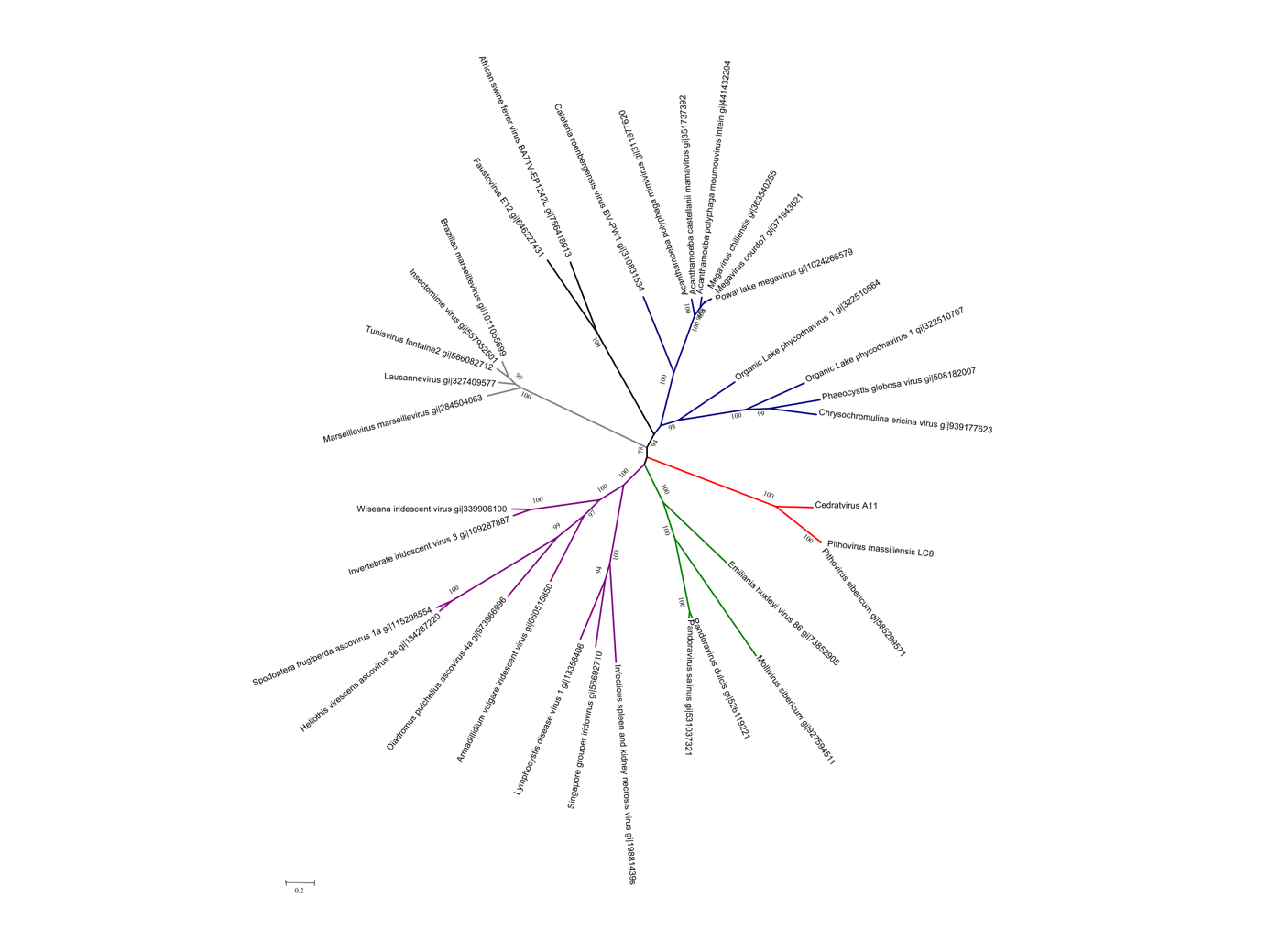


**Figure S2.** Unrooted tree based on DNA-dependent RNA polymerase II subunit 2 amino acid sequences; bootstrap values inferior to 0.5 (50%) were collapsed, branch length was applied; colors indicate family or viral groups: blue was used for *Mimiviridae* and an extended group including *Phaeocystis globosa virus*; purple for *Ascoviridae-Iridoviridae*; grey for *Marseilleviridae*; red for Cedratvirus and pithoviruses; green for *Phycodnaviridae*, pandoraviruses, and *Mollivirus sibericum* and black color for African swine fever virus and Faustovirus.


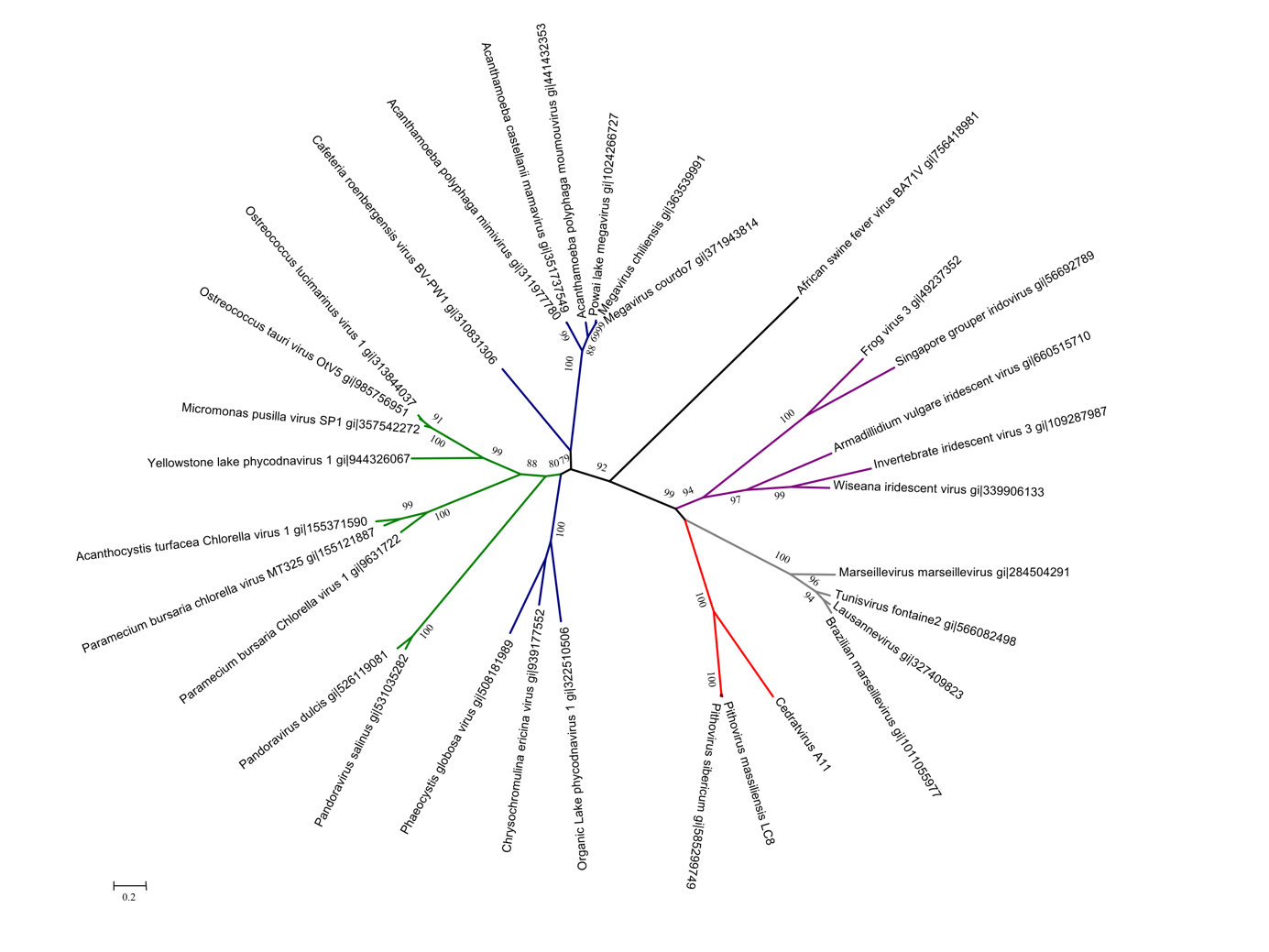


**Figure S3.** Unrooted tree based on VV-A18 helicase amino acid sequences; bootstrap values inferior to 0.5 (50%) were collapsed, branch length was applied; colours indicate family or viral groups: blue was used for *Mimiviridae* and an extended group including *Phaeocystis globosa virus*; purple for *Ascoviridae-Iridoviridae*; grey for *Marseilleviridae*; red for Cedratvirus and pithoviruses; green for *Phycodnaviridae*, pandoraviruses, and *Mollivirus sibericum* and black color for African swine fever virus and Faustovirus.


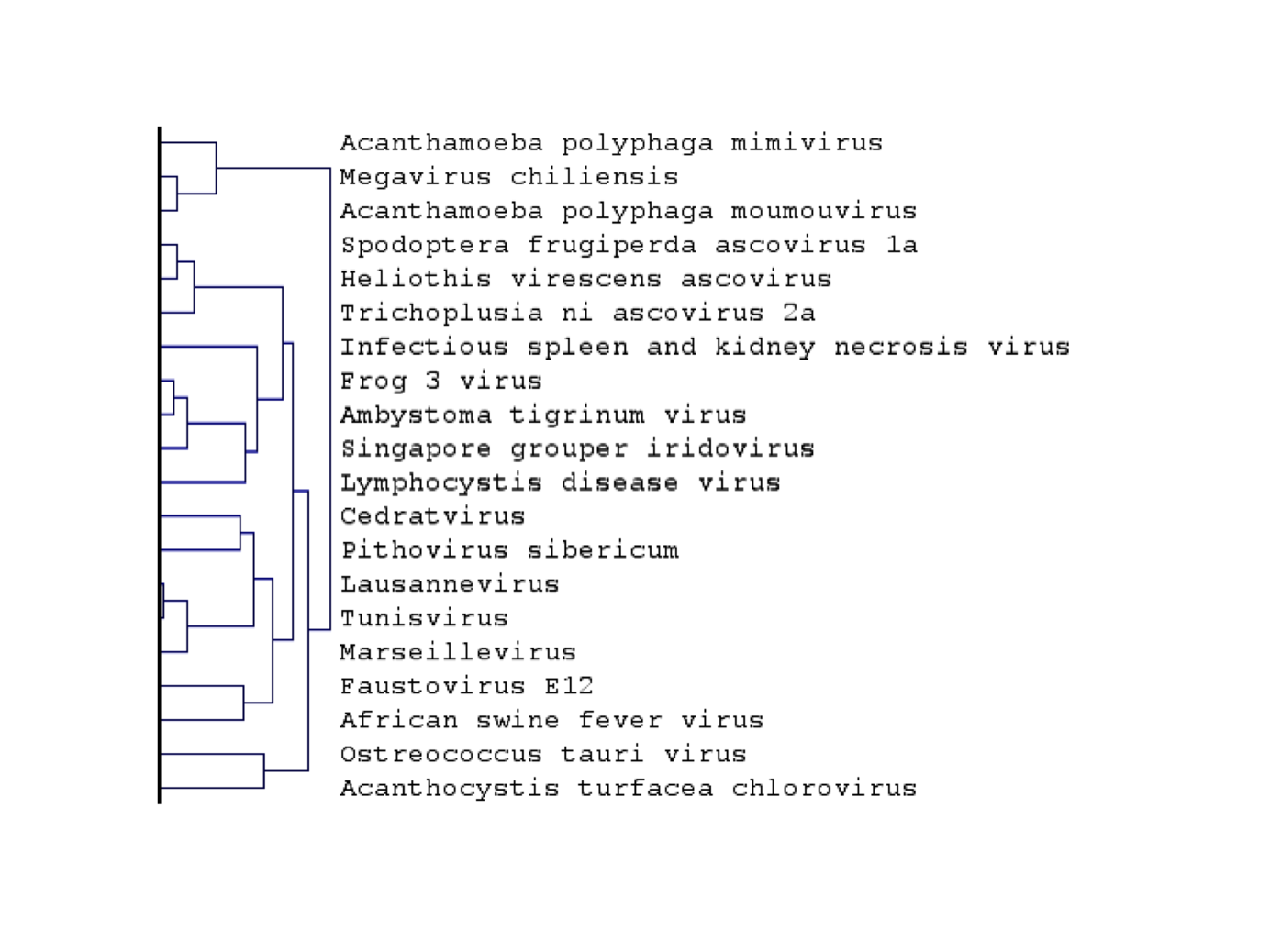


**Figure S4.** Cladogram based on NCVOGs; including Cedratvirus, based on the matrix of the presence/absence on the nucleo-cytoplasmic virus orthologous groups of proteins (NCVOG).
